# Understanding Heritable Variation Among Hosts in Infectious Diseases Through the Lens of Twin Studies

**DOI:** 10.3390/genes16020177

**Published:** 2025-02-01

**Authors:** Maria K. Smatti, Hadi M. Yassine, Hamdi Mbarek, Dorret I. Boomsma

**Affiliations:** 1 Biomedical Research Center, QU Health, Qatar University, Doha 2713, Qatar; hyassine@qu.edu.qa; 2 Qatar Precision Health Institute, Qatar Foundation, Doha 5825, Qatar; hmbarek@qf.org.qa; 3 Complex Trait Genetics, Vrije Universiteit Amsterdam, 1081 HV Amsterdam, The Netherlands; di.boomsma@vu.nl; 4 Amsterdam Public Health (APH) Research Institute, 1081 HV Amsterdam, The Netherlands; 5 Amsterdam Reproduction and Development (AR&D) Research Institute, 1081 HV Amsterdam, The Netherlands

**Keywords:** twin model, infection, host genetics, vaccine response

## Abstract

Genetic factors have been hypothesized to contribute to the heterogeneity in the response to infectious diseases (IDs). The classical twin design provides a powerful tool to estimate the role of genetic contributions to variation in infection outcomes. With this design, the impact of heritability on the proneness as well as infection- and vaccine-induced immune responses have been documented for multiple infections, including tuberculosis, malaria, leprosy, otitis media, polio, mumps, measles, rubella, influenza, hepatitis B, and human papillomavirus infections, and recently, SARS-CoV-2. The current data show the heritable aspect in nearly all infections considered. In this contribution, we review and discuss human twin studies on the heritability of host characteristics in liability and response to IDs. This review emphasizes the importance of considering factors such as sex, disease stages, and disease presentation when assessing heritability and argues that the classical twin design provides a unique circumstance for exploring the genetic contribution as twins share levels of maternal antibodies, ancestral background, often the dates and number of vaccine doses, differences in vaccines’ manufacturing and storage, age, family environment, and other exposures. Additionally, we highlight the value of twin studies and the usefulness of combining the twin model with contemporary genomics technologies and advanced statistical tools to grasp a comprehensive and nuanced understanding of heritability in IDs.

## 1. Introduction

The notion that human genetics modulates our interaction with pathogens can be traced back to the start of the 20th century. Around that time, plant, mammalian, and human geneticists suggested that infectious diseases (IDs) had a strong host genetic component [1]. In humans, the clustering of infections among families also pointed to the potential heritability in IDs [2], although family members often share their environmental exposures, as well as their genotype. The wide spectrum of clinical manifestations in different individuals exposed to the same infection, despite the lack of demographic differences, also raised the question of the role of host genetic factors in shaping viral/bacterial pathogenesis. Given their typically similar environmental conditions and exposures, combined with the differential similarity in their genomes, studies in monozygotic (MZ) and dizygotic (DZ) twin pairs have historically contributed to addressing these questions.

The value of the twin model in science and medicine dates back to 1875, when Francis Galton proposed to study twins as a test of the heredity hypothesis, although he did not realize at that time the distinction between the two types of twins [3,4]. Since 1924, when the first twin study, comparing similarities for the mole count (melanocytic nevus) of MZ and DZ twins, was published by Siemens [5], the classical twin design has been applied to estimate the role of genetics and the environment in explaining population variation in continuous traits and disorders.

**Figure 1:**
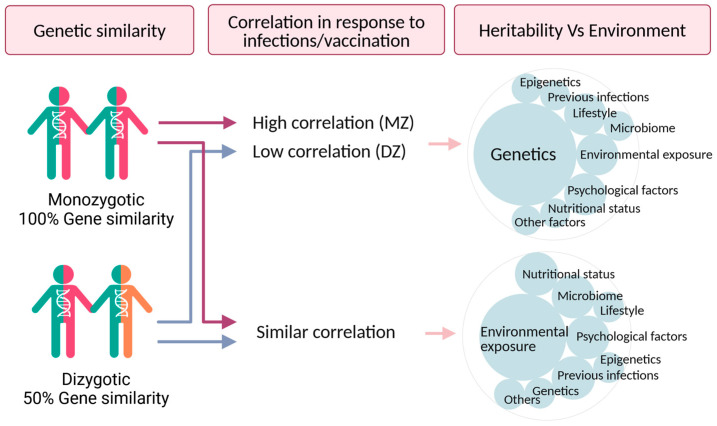
Genetic similarity and twin resemblance in response to infections/vaccinations. This figure demonstrates the association between genetic similarity and the correlation in immune responses in monozygotic (MZ) and dizygotic (DZ) twins. MZ twins, with almost 100% of genetic similarity, exhibit higher resemblance in their immune responses compared to DZ twins, who share 50% of their segregating genes. Low correlations in DZ twins compared to MZ twins suggest that genetic factors play a role in determining the trait, as the environmental influences would affect both twin types equally while similar correlations in MZ and DZ twin pairs indicate that shared environmental factors (e.g., lifestyle, microbiome, and previous infections) play a role in shaping the immune response. A combination of genetic and shared environmental influences as an explanation of resemblance would be indicated when the correlation in MZ pairs is less than twice the DZ correlation. (Figure created using Biorender).

The logic behind the twin design is that MZ twins share all, or nearly all, of their DNA, whereas DZ twins share, on average, 50% of their segregating genes, like other full siblings. Both identical (MZ) and fraternal (DZ) twins share prenatal environmental exposures, and their later environmental similarity in exposures includes, but is not limited to, age, nutrition, neighborhood, and socioeconomic status. Hence, any differences observed in the resemblance between the infection or vaccination outcomes in DZ compared to MZ twin pairs are consistent with a genetic influence on the outcome trait (Figure 1).

The statistical measures of association for cross-classification in twin pairs, i.e., the association of a trait in one twin with the trait in its co-twin, are called concordance rates (CRs) [6] or tetrachoric correlations for binary traits and Pearson or intraclass correlations for continuous traits. These measures are calculated for MZ and DZ pairs, and the comparison between the two values will suggest a genetic effect when MZ twin pairs show a higher resemblance than DZ twin pairs. For CR, where the proband-wise concordance rate is preferred over the pairwise concordance [7], the associations for dichotomous traits are represented on the observed scale (e.g., affected/unaffected). For estimating the heritability (*h*^2^), the observed bivariate dichotomous data are analyzed on the scale of liability, where a threshold divides the underlying liability scale into values that represent affected and unaffected groups [8].

Heritability refers to the proportion of variability in a phenotype that results from genetic differences between individuals, and a first estimate may be obtained by doubling the difference between the MZ and DZ correlations [9]. This gives a value for heritability that ranges from 0 to 1 (or 0 to 100%), with a low value indicating a smaller genetic contribution compared to the environmental component and a high value of a larger genetic influence. Because DZ twins are born at the same time and, like MZ twins, share prenatal conditions and post-natal exposures, they serve as the control group for MZ twins. Generally, the expectation for the MZ correlation equals *h*^2^ + *c*^2^, and for DZ twins, ½*h*^2^ + *c*^2^, where *h*^2^ (heritability) stands for the variance explained by genetic factors and c^2^ stands for the variance explained by a common environment that is shared by twins. Based on this approach, twin studies have shown that resemblance for multiple IDs is greater for MZ than for DZ twins, pointing to a, sometimes substantial, heritability.

In this review, we describe the contributions of twin studies to exploring the host genetics of IDs, as well as some limitations, and the value of this unique model in the current era of advanced genomics.

## 2. Twin Studies of Susceptibility and Response to Infections

The classical twin model has been applied to the field of IDs since the 1930s. Notably, the twin design investigated the genetic components in three main aspects: (1) Infection susceptibility and outcomes, including the spectrum of clinical and immunological responses. (2) The immune response to vaccination as well as vaccine effectiveness. (3) The heritability of general innate and adaptive immunity features. Table 1 summarizes twin studies on the susceptibility or response to various IDs.

Earlier studies on host genetics focused on tuberculosis (TB). The idea of heritability in infections has long been considered in the medical field, as phthisis (TB) often appeared clustered within families. Hippocrates (460–377 B.C.) and others believed TB to be hereditary rather than infectious. However, a few hundred years later, Galen (129–210 CE) proposed it is contagious, which explained its spread pattern [2]. Although it is now recognized that TB and other infections are never inherited in the true meaning of heredity, the concept of “inheritance” was introduced again into the field of IDs in the context of inheriting host susceptibility or resistance. The first evidence that host genetic factors influence TB susceptibility came from twin studies, including some historical ones. In 1936, the heritability in the incidence of TB was investigated by observing 205 twin pairs, and higher concordance for TB was found in MZ (65%) than in DZ twin pairs (25%) [10]. Not only the incidence but also a consistent behavior following infection was observed between most of the adult identical twins compared to fraternal twins who exhibited considerable differences.

Interestingly, this observation was less likely among infantile and juvenile cases, where environmental factors might play a more decisive role. In another seminal study a few years later, Kallmann and Reisner reported similar findings on 308 twin pairs in the U.S., in which one twin member was a confirmed index TB case. The TB infection rate was 66.7% for MZ twins compared to 23% for DZ twins [11]. These early studies, as well as others, received criticisms regarding the method of ascertainment, including voluntary participation and self-selection in the study. To overcome these methodological problems, a subsequent well-designed survey conducted in 1963 re-tested Kallmann and Reisner’s conclusions after applying refinements of the methods [12]. Here, only twin pairs, one of which was known to be affected by TB, were contacted for inclusion. Out of 205 pairs of twins included in the study, 55 and 150 were MZ and DZ, respectively. The concordance for TB was 32.7% in MZ pairs compared to 14% in DZ pairs. A higher proportion of MZ pairs were exposed to a positive sputum and lived in the same house, suggesting that the higher concordance rate for TB among MZ twins might be attributed to their greater physical contact, greater incidence of monozygotic index cases with a positive sputum, and probably by a greater number of susceptible females among the MZ twin pairs as well. These interesting data were re-analyzed by multiple groups that reached different conclusions. In 1978, Comstock concluded that concordance for TB was significantly higher among MZ (32%) than DZ (14%) twin pairs [13]. This finding highlighted that inherited susceptibility is an important risk factor for TB. Around three decades later, van der Eijk et al. re-analyzed the Prophit Survey again, reconsidering the role of environmental factors in determining the concordance rate of TB [14]. In this re-analysis, researchers initially found similar results to Comstock’s results, but when accounting for effect modifiers, genetic susceptibility was suggested to be a masked variance in exposure risk [15]. Environmental factors related to the intensity and duration of exposure to tubercle bacilli, including sputum smear positivity, zygosity (physical proximity between twins), and living together at the time of contagious TB in the index case, outweighed hereditary factors in TB concordance [14].

Twin and family studies also provided the first glimpse into the role of host genetics in hepatitis B virus (HBV) outcomes. Lin et al. reported significant differences in the concordance for HBV persistence between MZ and DZ twins and between MZ twins and controls in 391 twin pairs and 375 pairs of age–sex-matched singletons [16]. MZ twins showed a 35% concordance for chronic HBV, compared to 4% in DZ twins. A smaller study (*n* = 20 twin pairs) also reported high concordance among MZ compared to DZ twins in the rate of infection, clinical phenotype, and serological patterns following HBV infection [17]. A report aimed at exploring to what extent the immune profile depends on environmental and genetic factors reported a heritable bias in T-cell receptor (TCR) repertoire features from 10 identical twins [18]. A considerable level of similarity between twins was observed in the average TCR Vβ expression regardless of the consistency in HBV chronicity. However, specific CDR3 patterns and frequencies were associated with disease prognosis. This highlighted the value of exploring the CD8+ T-cell repertoire of identical twins with concordant chronic infections, considering its potential prognostic and immunotherapeutic applications [18].

**Otitis media** (OM) is one of the most common childhood infections that primarily occurs coincident with viral upper respiratory tract infections and/or bacterial infections [19]. This includes respiratory syncytial virus, rhinovirus, adenovirus, parainfluenza, and coronavirus, as well as *Streptococcus pneumoniae*, *Moraxella catarrhalis*, and non-typeable *Haemophilus influenzae*. Twin studies consistently confirmed a contribution of genetic factors in developing episodes of middle ear effusion (MEE) and acute otitis media (AOM). One of the earlier studies was conducted on a cohort of 2750 Norwegian twin pairs born between 1967 and 1974, who had repeated measures on recurrent childhood otitis media [20]. This study showed that among females, variation in acquiring ear infections was primarily explained by genetic heritability (74%) and individual environmental factors (26%). On the other hand, in males, 45% of the variation was explained by genetic factors, compared to 29% and 26% for common familial and individual environmental effects, respectively. This study highlighted the possible sex differences in the contribution of genetics in otitis media. In a subsequent analysis, Kvestad et al. utilized the data from Norwegian twins born between 1967 and 1979 (*n* = 4247 twin pairs) to estimate the relative impact of genetic and environmental factors in otitis media with particular attention to sex differences [21]. This analysis indicated that additive genetic effects explained 72% and 61% of the variance in males and females, respectively, confirming firstly the substantial genetic influence on liability to otitis media and secondly showing that a model specifying equal heritability estimates for males and females yielded an almost equivalent fit, thus questioning a sex difference in genetic architecture. Time with and episodes of MEE and AOM were also strongly associated with a genetic component, as shown in a study on a clinically well-characterized twin and triplet cohort [22]. A high heritability estimate of time with middle ear effusion reaching 0.73 was reported during the first 2 years of life. In addition, discordance estimates for three or more episodes of MEE and an episode of AOM were much lower among MZ compared to DZ twins (0.04 compared to 0.37 and 0.04 compared to 0.49, respectively). The strong genetic component appeared to attenuate during a 5-year follow-up, but its cumulative effect remains significant after 5 years [23].

In addition to the time and number of OM infections, another key finding from twin studies is the variable contribution of genetics on acute and chronic conditions. A large longitudinal study (*n* = 1373 twin pairs) demonstrated the difference in heritability across different OM phenotypes [24]. AOM infections showed lower heritability (0.57) and a higher shared environment (0.18) compared to chronic airway blockage (0.72 heritability and 0.10 shared environment). The higher heritability of chronic compared to AOM could be attributed to the involvement of genes related to host immune and inflammatory responses, mucin production, mucociliary transport, and the development of the ME cavity and craniofacial structure as reported from human and mouse studies [25]. Collectively, these reports confirmed the heritable aspect of otitis media, with the possible effect of sex differences, phenotypic stages, and disease presentation on heritability estimates.

The clinical presentation of malaria was also shown to be under genetic control. In a longitudinal study of malaria morbidity that included 258 twin pairs, it was found that fever in malaria is genetically influenced [26]. Genetic factors regulated the immune response to *Plasmodium falciparum* in a twin study on 519 twin adult and child twin pairs, where heritability of different plasma antibody isotypes and subclasses reached 48%, with IgG4 showing higher heritability among children [27]. Importantly, genetic factors that regulate antibody isotypes and subclasses in response to malaria antigens were mainly attributable to undefined non-HLA-linked genes. These results point to additional studies to discover the responsible genetic loci.

Genetic factors substantially modulate the innate anti-inflammatory cytokine profile and influence the development of fatal meningococcal disease [28]. In 190 first-degree relatives of 61 patients with meningococcal disease and 26 MZ twins, the heritability of TNF and IL-10 production was 60% and 75%, respectively. Moreover, families exhibiting high IL-10 production or low TNF production had an increased risk of the fatal outcomes by 10- and 20-fold, respectively. This shows the value of investigating the role of host genetics on different aspects of the immune system to understand the correlation with infection outcomes. Studying the genetic underpinnings of the immune response is a feasible avenue of scientific inquiry as it is restricted to research involving individuals who have certainly contracted an infectious agent. On the other hand, the susceptibility to IDs is more challenging as it depends on the likelihood of becoming infected following exposure to a pathogen. This, in turn, depends on multiple factors, including the pathogen infective dose and the host protective measures.

Although not explored extensively for each pathogen, the heritable contribution to the development and severity of multiple other infections was demonstrated in earlier and recent twin studies. For instance, a report in 1951 investigated the twin pair concordance of paralytic poliomyelitis to answer questions raised during that period (1939–1950) on the host genetics of acute poliomyelitis [29]. This report investigated the heritability in 46 families with twins or triplets, of whom one or more had paralytic poliomyelitis. Families with twins were identified in an unselected series of 3890 reported cases of poliomyelitis in North Carolina (USA). The authors show that the frequency of twins ascertained this way did not differ from the corresponding frequency for the total United States population. A significant difference in the concordance between MZ and DZ twin pairs with regard to paralytic poliomyelitis was reported (36% vs. 6%, respectively). Obviously, this finding indicated that genetics, at least partially, contributes to susceptibility to poliomyelitis.

High concordance among MZ twins in the susceptibility to other IDs has been reported by multiple subsequent reports. In 1983, a study on endouterine cytomegalic viral infection in twins reported concordance in one case of MZ twins while discordant in two cases of DZ twin pairs [30]. A study of six IDs (measles, mumps, chickenpox, German measles, scarlet fever, and whooping cough) conducted in 1984 on a sample of 656 twin pairs estimated heritability as 86% for measles to a 100% environmental component for scarlet fever [31]. This study emphasizes that a genetic contribution to disease susceptibility is variable between different IDs.

The proband-wise concordance rate for ***Helicobacter pylori*** (*H. pylori*) infection was higher in MZ twin pairs (81%) compared to DZ twin pairs (63%), as reported in a twin study which included entries for about 25,000 twin pairs born in Sweden. The study could compare twin pairs reared apart and reared together. The proband-wise concordance rates for *H. pylori* infection in 124 twin pairs reared apart were 82% and 66% for MZ and DZ pairs, respectively (*p* = 0.003). The correlation coefficient was 0.66 for MZ twins reared apart, and this probably gives the best single estimate of heritability. The model-fitting analyses revealed a similar heritability estimate of 57%, while the remaining variance was explained by a shared rearing environmental (20%) and non-shared environmental effect (23%) [32].

**Leprosy infection** was highly concordant among MZ (60%) compared to DZ twins in a 1973 study that involved 102 twin pairs in India [33]. It is important to note that both the characteristics of the population and the time at which it was studied may influence the heritability estimates. For instance, a twin study on the heritability of **tonsillectomy** found a higher concordance in MZ compared to DZ twins [34]. However, the influence of genetics and environmental factors varied considerably between cohorts according to the year of birth. During the 1950s, when tonsillectomy was fashionable, heritability was only 29% compared to 60% of the contribution by environmental factors. In contrast, when the number of tonsillectomies started to decline in the 1960s, genetic factors accounted for up to 82% of the variance compared to only 10% explained by the environmental impact.

Twin studies on IDs continued to provide insights into heritability, with multiple studies on various infections conducted in the last decade. Hwang et al. assessed the heritability of **infectious mononucleosis** (IM) caused by Epstein–Barr virus (EBV) infection in 6926 MZ and DZ twin pairs [35]. The concordance for IM in MZ twins was twice that of DZ twins (12.1% vs. 6.1%, respectively), and the hazard ratio of MZ compared to DZ unaffected co-twins of cases was 1·9. This study stressed the importance of looking at the genetic contribution in EBV infection, as this virus is highly prevalent and linked to chronic conditions such as EBV-positive Hodgkin’s lymphoma and multiple sclerosis. The study also suggested that “understanding the nature of this genetic susceptibility may provide valuable clues to the etiology of these and other important chronic diseases”.

**Human papillomavirus** (HPV) is another widely circulating oncovirus that has been studied by the twin model. Familial aggregation for cervical cancer spotlighted the potential role of genetics in disease development [36]. Data from MZ and DZ twin pairs and their female relatives were analyzed to explore the influence of heritable and environmental elements on cervix smear abnormalities [37]. MZ twins demonstrated a stronger correlation of cervix smear abnormalities (0.37) compared to DZ twins (0.14). Genetic factors explained 37% of the familial clustering of the abnormal cervix smear, with the largest proportion of the variation being due to environmental factors.

Recently, the heritability of **SARS-CoV-2** clinical manifestations among twins was estimated in the TwinsUK registry participants [38]. Heritability ranged from 19% to 49% for different SARS-CoV-2 symptoms, including fever, fatigue, diarrhea, and others. For a predicted COVID-19 phenotype, 31% of the variance was attributable to genetics. A study that investigated COVID-19 outcomes in 10 pairs of young twins reported higher concordance in the MZ twin pairs, supporting a complex multifactorial predisposition to SARS-CoV-2 [39]. The same group investigated the case of a young adult MZ twin pair after simultaneous critical COVID-19 that required oxygen support, despite their young age and good health conditions [40]. While the MZ twin brothers shared identical genetic mutations that might contribute to severe COVID-19, their individual disease progression varied, with one of the twins requiring extended hospitalization time and experiencing post-COVID syndrome. This underscores the significance of other immune response factors in addition to genetic factors in COVID-19 presentation and course [40]. Rupp et al. estimated variances explained by genetic, shared, and individual environmental factors in the development of physical and psychological symptoms following SARS-CoV-2 infection in 10 adult twin pairs (5 MZ and 5 DZ twins) [41]. Interestingly, a high heritability was estimated for mental impairment and general fatigue, while symptom burden and other fatigue symptoms were more influenced by the non-shared environment, highlighting the role of the environment–gene interplay in SARS-CoV-2 somatic and psychological symptoms [41].

**Table 1:** Twin studies on the susceptibility or response to various IDs.

	Infection/Condition	Study Title	Year	Sample Size	Main Findings	Ref.
1	Tuberculosis	Der erbeinfluss bei der tuberkulose (zwillingstuberkulose II)	1936	205 twin pairs	Concordance: MZ: 65%–DZ: 25%	[10]
		Twin Studies on the Significance of Genetic Factors in Tuberculosis	1943	308 twin pairs	Concordance: MZ: 66.7%–DZ: 23%	[11]
		Tuberculosis in Twins	1963	205 twin pairs	Concordance: MZ: 32%–DZ: 14%	[12]
		Tuberculosis in Twins: A Re-Analysis of the Prophit Survey	1978	205 twin pairs	Concordance: MZ: 32.7%–DZ: 14%	[13]
2	Hepatitis B virus	Hepatitis B virus markers in Chinese twins	1989	391 twin pairs 375 pairs matched singleton	Concordance: MZ: 35%–DZ: 4	[16]
		The primary comparative analysis between the host genetic factors and their relationships with clinical phenotype of HBV infected twins	2004	20 pairs (HBV-infected and high risk twins)	Concordance: Significant difference in concordance between MZ and controls, and between MZ and DZ.	[17]
3	Otitis media	Distribution and Heritability of Recurrent Ear Infections	1997	2750 twin pairs	Heritability: 0.74 in females and 0.45 in males	[20]
		Otitis media: genetic factors and sex differences	2004	4247 twin pairs	Heritability: 72% in females and 61% in males	[21]
		The heritability of otitis media: a twin and triplet study	1999	140 twin pairs	Heritability: 0.73 0.64 in males and 0.79 in females	[22]
		Heritability of Symptom Domains in Otitis Media: A Longitudinal Study of 1373 Twin Pairs	2002	1373 Twin pairs	Correlation: MZ: 0.9–DZ: <0.65 Heritability: 0.49–0.66, and 0.71, for different age groups	[24]
4	*Plasmodium falciparum*	Genetic regulation of fever in *Plasmodium falciparum* malaria in Gambian twin children	1995	258 twin pairs	Fever in malaria is genetically regulated	[26]
		Heritability of antibody isotype and subclass responses to *Plasmodium falciparum* antigens	2009	519 twin pairs	Heritability: up to 48% for different plasma antibody isotypes and subclasses	[27]
5	Meningococcal disease	Genetic influence on cytokine production and fatal meningococcal disease	1997	190 first-degree relatives 26 MZ twins	Heritability: MZ: 0.60 for TNF and 0.75 for IL-10	[28]
6	Pliomyelitis	A twin-family study of susceptibility to poliomyelitis	1951	46 families who had twins or triplets	Concordance: MZ: 36%–DZ: 6%	[29]
7	Cytomegalovirus	Neonatal cytomegalic inclusion disease in a set of twins one member of whom was a hydropic stillbirth the other completely uninfected	1983	3 twin pairs	Concordanct in one case of MZ twins while discordant in two cases of DZ twin pairs	[30]
8	Multiple infections	Heredity and IDs: a twin study	1984	656 twin pairs	Heritability: variable 86% of measles 0% scarlet fever	[31]
9	*Helicobacter pylori*	*Helicobacter pylori* infection: genetic and environmental influences. A study of twins	1994	269 pairs: 36 MZ pairs reared apart 64 MZ pairs reared together 88 DZ pairs reared apart 81 DZ pairs reared together	Correlation: MZ (reared apart): 0.66 Concordance: MZ: 81%–DZ: 63% MZ reared apart: 82% DZ reared apart: 62% Heritability: 0.57	[32]
10	Leprosy	A Twin Study on Leprosy	1975	102 twin pairs	Concordance: MZ: 60%	[33]
11	Tonsillectomy	Iatrogenic influences on the heritability of childhood tonsillectomy: cohort differences in twin concordance	1991	NA	Heritability: In the 1950s: 29% Early 1960s: 82%	[34]
12	Epstein–Barr virus	Evidence of genetic susceptibility to infectious mononucleosis: a twin study	2012	6926 twin pairs	Concordance: DZ: 12.1–MZ: 6.1	[35]
**13**	Human papillomavirus	Cervix smear abnormalities: linking pathology data in female twins, their mothers and sisters	2011	2020 twins 671 sisters of twins 416 mothers of twins 71 female spouses of male twins.	Correlation: MZ: 0.37–DZ and other first-degree relatives: 0.14 Heritability: 37%	[37]
**14**	Death due to infections	Genetic and Environmental Influences on Risk of Death due to Infections Assessed in Danish Twins, 1943–2001	2010	44,005 same-sex twin	Concordance: MZ vs. DZ: 9% vs. 0%, 10% vs. 3%, and 19% vs. 15%	[42]
**15**	SARS-CoV-2	Self-Reported Symptoms of COVID-19, Including Symptoms Most Predictive of SARS-CoV-2 Infection, Are Heritable	2020	3261 twins	Heritability: 19% to 49% for different SARS-CoV-2 symptoms	[38]
		COVID-19 in twins: What can we learn from them	2021	10 twin pairs	Higher concordance rates in the MZ twin pairs	[39]

NA: Data not available.

## 3. Twin Studies of Response to Vaccination

**Figure 2:**
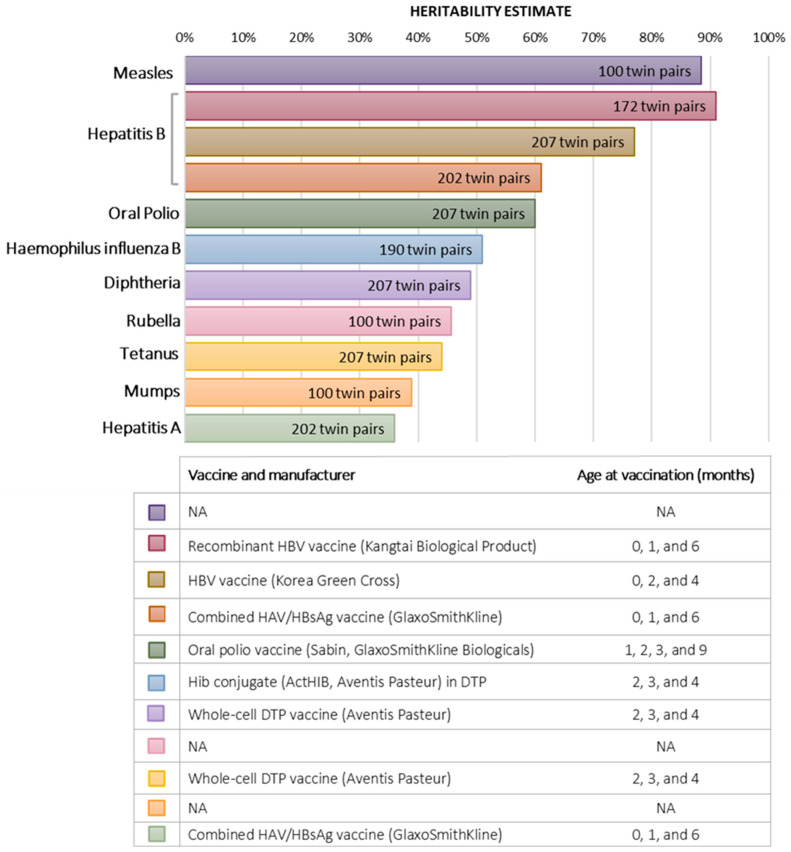
Heritability of antibody response following vaccination against different infectious diseases. NA: Data not available.

The extent to which genetic variation influences vaccine effectiveness or failure has long been a topic of inquiry [43]. Twins share the levels of maternal antibodies, ancestral background, often the dates and number of vaccine doses, differences in vaccines’ manufacturing and storage, age, family environment, and others [44]. Sharing all these non-genetic factors provides a unique circumstance for exploring the magnitude of genetic contribution to vaccine response while minimizing the influence of confounding factors. Twin studies on the response to immunization explored the heritability of the immune response and adverse events following vaccination against a wide range of pathogens. This includes but is not limited to *mycobacterium tuberculosis* (Bacillus Calmette–Guérin, BCG), polio, diphtheria, pertussis, tetanus, measles, rubella, mumps, hepatitis B, malaria, and *Haemophilus influenza* type B (Hib) infections (Figure 2).

The Gambia Twin Study Group reported high heritability for antibody responses to hepatitis B (77%), tetanus (44%), oral polio (60%), and diphtheria (49%) vaccines in 207 Gambian twin pairs aged five months [45]. IFN-γ and IL-13 cytokine responses to tetanus, pertussis, and BCG vaccines also showed a variable heritability (39–65%). In contrast, a follow-up study (*n* = 210 Gambian twin pairs recruited at birth), one month after immunization, found no significant contribution of genetic factors to anti-tetanus antibodies nor the total IgG levels at 12 months of age [46]. However, genetic factors controlled measles antibody responses in 12-month-old infants. This study indicated that genetic determinants control the vaccine antibody response at an early phase of life, while environmental factors mainly modulate antibody persistence and avidity maturation. Tan et al. have also reported the substantial role of genetics on the antibody response to the measles vaccine in a study conducted on a total of 100 twin pairs in 2001 [47]. The study included subjects aged 2 to 18 years who had received at least one dose of each childhood vaccine and were young enough to remain living together. A heritability of 88.5% was found for measles, while mumps and rubella showed heritabilities of 38.8% and 45.7%, respectively.

Hepatitis B vaccine failure is estimated to be 5–10% [48]. This is a strong reason to study the influence of genetics on the immune responsiveness to the HBV vaccine. Newport et al. showed that the heritability of anti-HB’s response reached 77% in the Gambian study twins [45]. Another study on 202 twin pairs, who were administered a combined recombinant hepatitis B surface antigen/inactivated hepatitis A (HAV) vaccine, showed high heritability of anti-HBs (61%) [49]. However, the heritability of the antibody response to the HAV vaccine was much lower (36%). This study also estimated the contribution of the DRB1* locus (MHC gene) to HBsAg heritability, which was estimated to be 0.25 (out of 0.61). The remaining heritability of 0.36 was attributed to non-MHC genes. Furthermore, the ACC haplotype (−1082, −819, and −592) in IL-10—a central immunoregulatory cytokine—has shown to strongly influence anti-HB’s production. In contrast, anti-HAV production was suppressed by the presence of the −1082A allele compared to individuals homozygous for the −1082G allele. Importantly, 27% of the genetic contribution in anti-HB’s antibody response was due to the shared IL-10 promoter haplotypes. More recent reports confirmed these findings. In 2013, a study conducted on 172 Chinese twin pairs to understand the poor response to HBV vaccination found that genetic factors contribute to 91% of the vaccine response compared to perinatal environmental factors [50].

Vaccine failure has been also reported in Hib vaccines. This disease was once the global leading cause of multiple complications such as bacterial meningitis, pneumonia, and epiglottitis in children below five years of age [51]. Although the introduction of the Hib vaccine has reduced the infection burden, the variability in vaccine efficacy suggested a genetic influence. A twin study on 43 MZ and 147 DZ twin pairs estimated heritability at 51% in the antibody responses to the Hib conjugate vaccine [51]. This indicated that host genetic variations might underlie the difference in the immune response to the Hib vaccine and may contribute to conditions of vaccine failure. However, the particular genes that are involved in this heritability remained undetermined.

One of the main obstacles that led to the delay in developing a successful malaria vaccine was the heterogeneity in response to malaria antigens. Hence, several genetic studies, including twin-based studies, have focused on delineating the genetic control of the humoral and cellular immune responses to malaria antigens. In a population of 271 adult twin pairs in Gambia, Jepson et al. reported that the regulation of the immune response to a variety of malaria antigens is under the genetic control of the MHC and non-MHC genes [52]. This study has also revealed that heritable factors contribute significantly to cellular immune responses to the purified protein derivative (PPD) of *Mycobacterium tuberculosis*. In addition, it has been demonstrated that memory T-cell responses to secreted mycobacterial antigens (85-kDa antigen complex, “short-term culture filtrate”, and peptides from the ESAT-6 protein), and to the 65-kDa heat shock protein, are all under a strong genetic control [53]. A delayed hypersensitivity response to intradermal tuberculin was also significantly influenced by genetic variance [53].

Somatic genetic rearrangement is a critical process in the adaptive immune system that leads to the elicitation of a highly diverse repertoire of antibody-encoding sequences and TCRs that can recognize a large number of infectious agents. Notably, human immunity is substantially influenced by the germ-line genome in addition to the somatic genetic rearrangements [54]. Specifically, it has been previously shown that the germ-line genome sequence affects the early generation and selection of antibody and TCR repertoires [55]. Nonetheless, it is unclear whether this genetic effect is also extended to the clonal B-cell responses to a particular pathogen or vaccine. Wang et al. took advantage of the unique similarity in twins’ genomes to investigate the human B-cell responses that are mostly controlled by the germ-line genome [56]. Analysis of 134,000 antibody heavy chain sequences from four pairs of adult identical twins vaccinated with the varicella zoster vaccine (VZV) showed a high correlation in the antibody gene segment (V, D, and J) usage, junctional features, and mutation rates in their antibody pools. However, the B-cell clones used in acute responses to VZV vaccination were largely distinct in each individual. This indicates that the germ-line genome influences the overall B-cell repertoire, yet, following the exposure to antigen stimuli such as vaccination, individual-specific factors dominate [56].

Overall, these findings underscore the importance of the classical twin model in estimating overall, or broad-sense, heritability and in designs that combine with genotyping, partially explaining the influence of specific genes on immune responses to vaccination. Studies examining genetic polymorphisms at the whole-genome level and with a broad perspective are crucial for pinpointing the precise contribution of individual genes to vaccine response.

## 4. Twin Studies of General Immune Response

Multiple earlier reports quantified the heritability of immune traits. In an extended twin study, cytokine production was assessed in 42 MZ pairs, 52 DZ pairs, one trizygotic triplet, 33 single twins, and 83 additional siblings [57]. Interestingly, for all cytokines analyzed, more than 50% of the variance was genetically explained. IL-1ra and TNF-α demonstrated the lowest heritability estimate (53%), while the estimates for IL-6 and IL-10 were 57 and 62%, respectively. Moreover, IL-1beta showed the highest heritability (86%). These findings provided strong evidence for the genetic basis of inflammation and the innate immune response. This also opened the door for subsequent work towards locating novel genetic markers that are in control of the variability in immunological phenotypes.

Roederer et al. performed comprehensive immunophenotyping in 669 female twins, where 78,000 immune traits were analyzed [58]. This study was based on an approach that takes advantage of the twin model to pre-specify and prioritize independent immune traits for genome-wide association analysis and replication. The top 151 heritable immune traits (with a heritability up to 96%) were included in the GWAS. Multiple associations with canonical traits of the main immune cell populations were found. For example, FCGR2A variants were linked to numerous immune phenotypes such as the expression levels of various T-cell markers, including the CD27 and CD161 [58].

In another attempt to explore the role of genetic variation in modulating different immunological parameters, a combination of a classical twin study approach with the recent advances in immune monitoring technologies was utilized. Robin et al. performed a systems-level analysis on 210 healthy twins (8–82 years of age), and investigated 204 different immune traits [59]. This included 95 different cell populations, cellular responses to cytokine stimulation, serum levels of 51 cytokines, as well as cytokine, growth factors, and chemokine levels. It was found that 77% of these traits were dominated (>50% of variance) and 58% almost completely determined (>80% of variance) by non-heritable aspects. Although multiple immune cells demonstrated a strong heritability, such as naive T-cells, CD27-expressing T-cells, and central memory CD4+ T-cells, most cell populations were determined by non-heritable factors. Likewise, despite the high heritability observed for IL-12p40, G-CSF, GM-CSF, IFN-α, IL-6, and IL-7, many other serum cytokines did not appear to be heritable. Interestingly, this study has also highlighted immune traits that have high heritability, which declines with increasing age, such as levels of regulatory T-cells, and serum levels of the chemokine CXCL10. This could be mainly attributed to the repeated exposure to pathogens and other microbes in older age groups [59,60].

## 5. Twin Studies of Microbiome Composition

The human microbiota consist of an enormous range of microbial species primarily residing in the gut, where they engage in symbiotic interactions with the host. These interactions are crucial in maintaining health and, if disturbed, can lead to colonic diseases such as inflammatory bowel disease [IBD] or systemic disorders [61]. Alteration in the airway microbiota composition, on the other hand, may lead to respiratory diseases such as sinusitis, chronic obstructive pulmonary disease (COPD), and asthma [62]. While a range of environmental factors (e.g., diet and antibiotics) are accepted as key microbiome modulators, the degree to which host genetic variation can affect the microbial communities in humans still lacks a definitive answer.

The twin model has provided a framework for investigating the association between host genetics and the microbiome [63]. By using culture-based and fecal 16S rRNA gene amplicon fingerprinting methods, these studies suggested that parts of the microbiome might be heritable. Stewart et al. reported significant fecal microbiome differences between identical and fraternal twins, suggesting a genetic influence [64]. In another study, comparing the profiles of fecal bacterial 16S rDNA amplicons from adults with varying degrees of genetic relatedness revealed a higher similarity in monozygotic twins than in unrelated individuals [65]. Moreover, the fecal bacterial profiles of marital partners sharing the same environment showed lower similarity [65]. Likewise, a ribosomal DNA microarray-based study showed that intestinal microbiota assembly during the first year of life followed a similar pattern in a pair of DZ twins compared to unrelated infants (*n* = 12) [66]. However, these studies were limited by the insufficient sample sizes. Later twin studies with larger cohorts utilized 16S rRNA gene sequencing and revealed a dominant role of environmental factors compared to host genetic factors [67,68], although other studies proposed a broader genetic impact. In a twin study (416 twin pairs) from the TwinsUK population, Goodrich et al. identified multiple microbial taxa whose abundances were influenced by host genetics [69]. Increasing the sample size in a subsequent study (1126 twin pairs) uncovered additional heritable taxa and showed that gene–microbe association involves genes related to diet, metabolism, olfaction, and defense [63].

The degree to which genetic and environmental factors influence the human microbiome remains unclear. Recent evidence from twin studies highlighted the predominant role of environmental factors over host genetics in defining the microbiota composition in twins [70]. Profiling the supragingival plaque microbiomes of DZ and MZ twins revealed that oral microbiome variances are attributed primarily to environmental (e.g., age and brushing habits) influence rather than host genetic variation [71]. Remarkably, the role of host genetics has been much less investigated for the oral microbiome when compared to the gut. Although the overall knowledge of the host genetics impact on the airway microbiome still lags behind that of the gut, evidence of host genetic influences on upper airway microbial composition and association with mucosal immunity pathways has been reported [62]. Importantly, understanding this intricate relationship between the host, microbiome, and immune response requires comprehensive approaches that can benefit from the twin model and advanced genomic technologies. This will reveal genetic variations that influence functional microbial products and mucosal immunity, a pivotal player in response to infection and mucosal vaccine efficacy. Ultimately, bridging these knowledge gaps can help design more effective strategies tailored to diverse genetic and microbiome profiles.

## 6. Advantages of the Twin Model in the IDs Field

Classical twin studies have traditionally allowed the disentanglement of genetic and environmental factors in variations of response to IDs. Including twin studies to the general question of the genomics of IDs offers several valuable advantages. The study of DZ and MZ twins provides an interesting method to estimate the influence of genetic and environmental factors in a controlled setting [6]. Additionally, considering that MZ twins share 100% of their genome, when reared apart, they provide an ideal opportunity to explore the effects of a shared environment versus shared genes in infection outcomes. Although this does not eliminate the differences in infection exposure (pathogen dose), it provides a model that provides a better control on other factors. Furthermore, through the precise matching for confounding variables (age, sex, and common environmental determinants), the twin model represents a flexible and feasible study design that can be further amended to numerous non-classical study designs [72]. Twins provide a perfect control of confounders that cannot be controlled by studies on non-siblings/non-twins and may have a substantial influence on the heritability estimate of IDs. For instance, twins have a higher probability of having a similar history of other infections, concomitant medications, immunization types and doses, diet, and many others. Controlling for all these non-genetic factors would be extremely challenging in other study designs. In addition, the availability of large, established registries worldwide also offers an advantage for conducting such a type of analysis.

Compared to other genomic studies where hundreds of thousands of non-related individuals are needed to study the role of genetics on a specific trait, twin and family studies may estimate heritability and genetic correlations in smaller samples. This can be of a particular benefit to the field of IDs when host genetics of significant, yet non-widely prevalent infections need to be explored. Moreover, the limited number of participants in twin studies also provides the opportunity for detailed phenotyping, including follow-up assessments, which is unfeasible to perform in large-scale population genetics studies.

## 7. Challenges for the Twin Model in the IDs Field

MZ twins may often live in closer proximity than DZ twins. This may, in fact, be an important issue for IDs research, where closer proximity translates into a higher chance of infection. Resemblance in infection among MZ twins may therefore be a consequence of proximity, rather than a consequence of genetic similarity that is influencing the susceptibility to that infection. Hence, the classical twin design may in this case overestimate the heritability of IDs. In addition, estimates of shared environmental influences may be related to parental genetic factors (e.g., heritable parental behavior shapes the shared home environment of the twins), and thus in part explain twins’ shared environment. This process gives rise to the “passive genotype (A)-shared environment (C) correlation” [73]. In sibling studies of siblings reared in the same household, this could lead to the overestimation of environmental and the underestimation of genetic influences. However, in the classical twin design, this A-C covariance is included in the estimate of the shared environmental effects (C variance). While this C variance is biased, the genetic variance is not in the classical twin design.

Another potential limitation currently is that despite the great advantages of the existence of large twin registries, many of these registries depend on the voluntary participation of twins, which might lead to recruitment bias as, for example, is seen in an overinclusion of MZ and female twins. Recruitment bias and non-representative sampling could lead to biases in heritability estimates [6], and here, possibilities to link register data on twinning status and health records may hold great promise. Importantly, despite the growing number of twin registries globally, African, Arab, Hispanic, and other non-European populations are still underrepresented in twin research, as are some age groups [74].

## 8. The Twin Model in the Modern Genomic Era

Historically, the field of host genetics in IDs has been enriched by insights provided by twin/family studies. Understanding the genetic architecture of predisposition to infections continues to develop in recent years through the revolutionary progress in genomics, and large-scale genotyping, along with advances in biostatistics and bioinformatics. GWASs are now extensively applied and multiple GWASs investigated the basis of host genetics in association with infection susceptibility or resistance.

These newer study designs have not diminished the value of twin/family studies. The shift from twin studies to the GWAS approach is not a replacement of an outdated method with a modern one [74]. Twin/family studies continue to be an essential tool along with the continuously emerging genomics tools, and the complementarity between both can be exceptionally potent [75], and a promising design for the future is the combination of the two approaches, as evidenced by the study of Roeder et al. [58].

Twin studies have sometimes been criticized for overestimating heritability due to the assumption of the equal sharing of the environment between MZ and DZ twins. Hwang et al. discuss the equal environment assumptions and conclude that the empirical evidence for its violation is very limited [76]. In addition, new methods have been proposed to test this assumption, based on Identity by Descent (IBD) sharing in DZ pairs. Heritability estimates obtained from GWAS data (*h*^2^*_SNP_*) also can be biased, due to factors that might also influence the heritability estimates from twin studies, such as population stratification or assortative mating [77,78].

The most frequently assessed single-ancestry cohort in twin studies and GWAS is the European ancestry (81%), which limits the transferability to other populations [79]. Although both genotyping and twin approaches can be applied to estimate heritability, the heritability obtained from GWAS data (*h*^2^*_SNP_*) does not necessarily explain that of twin/family data (*h*^2^) [9]. The difference in heritability could be explained by several factors, including the fact that most GWASs utilize SNP genotyping arrays, which capture large numbers of common SNPs, but are missing the impacts of rare variants that are not/poorly tagged in the array. Consequently, for the same trait, the heritability estimate obtained from GWASs can be much lower than the heritability estimated from twin studies, leading to the issue of ‘still missing’ heritability (*h*^2^–*h*^2^*_SNP_*) [9]. For instance, the heritability of an antibody response following measles vaccination was 88.5%, as estimated from a twin study, while a more recent GWAS on 935 individuals reported 49% heritability [47,80]. However, increasing sample sizes and improving the methodological tools would fill the gap in heritability estimates from twin and genotyping studies [74]. In one of the largest human genetic studies so far, Young et al. analyzed the genomic data of 5.4 million people to explain the missing heritability of height, a trait that was previously explained by only 5% of GWAS variants. In addition to finding the missing heritability of height, their findings reached ‘saturation’ among the European ancestry, where additional genetic studies are not expected to reveal any new information. This astonishing report indicated that combining the additive effects of tens of thousands of individual variants from a large cohort enables explaining the considerable variation in complex human phenotypes. Importantly, this encourages implementing the same approach to explain the heritability of other traits and disorders, including IDs.

Taken together, the advantages and limitations of twin studies and the GWAS approach highlight the value of combining multiple models for scientific design and discovery. The high statistical power and well-matched controls of twin studies, along with the advantages of GWAS that allow deep variants enrichment analysis and gene identification, can be incorporated to answer critical scientific questions related to IDs.

Other than serving as a complementary approach to GWAS, studies on twin and family members always serve as a primary exploratory tool. This is evidenced by earlier studies and in recent reports. For instance, twin studies estimated the heritability of clinical manifestations and outcomes of SARS-CoV-2, as explained earlier in this review [38,39]. In parallel, a huge consortium of researchers collaborated to conduct a large GWAS and meta-analyses to understand the common variants contributing to the susceptibility and severity of COVID-19 [81]. Whether this COVID-19 GWAS, which is the largest GWAS in history, can fully explain the heritability of COVID-19 from twin studies will be determined in the future.

## 9. Conclusions

Twin studies have been important contributors to knowledge regarding the role of genetics in the course of infection and vaccination, especially now that twin studies can take advantage of the advanced set of statistical models and tools to improve the design and analysis. In the coming years, it would be of great value to utilize the longitudinal phenotypic assessments and the available biological samples obtained as part of twin registries around the world to conduct advanced genomics studies in these twin families. This will help in understanding causal interactive processes [82] and identifying the molecular mechanisms underlying the genetics–pathogen–host interplay.
